# The challenges and lessons from a formative process and value-based evaluation of the wave 1 roll-out of the all Wales Diabetes Prevention Programme

**DOI:** 10.1186/s12889-024-19946-0

**Published:** 2024-09-13

**Authors:** Sharon N Parsons, Liv Kosnes, Pippa Anderson, Shaun RS Harris, Rhys Thatcher, Barbara Harrington, Jan Davies, Deborah Fitzsimmons, Stephen D Luzio

**Affiliations:** 1 Diabetes Research Group, Faculty of Medicine, Health and Life Science, Swansea University, Swansea, UK; 2 Swansea Centre for Health Economics, Faculty of Medicine, Health and Life Science, Swansea University, Swansea, UK; 3 Department of Public Health, Faculty of Medicine, Health and Life Science, Swansea University, Swansea, UK; 4 Diabetes Research Group Swansea Public Reference Panel, Faculty of Medicine, Health and Life Science, Swansea University, Swansea, UK; 5 Dept of Life Sciences, Aberystwyth University, Aberystwyth, UK; 6 Centre for Health Economics and Medicines Evaluation, Bangor University, Bangor, UK

**Keywords:** Type 2 diabetes prevention, Prediabetes, Prevention and control, Programme evaluation

## Abstract

**Background:**

The All Wales Diabetes Prevention Programme (AWDPP) is a Wales wide, public health initiative designed to systematically identify adults at risk of developing type 2 diabetes and offer a 30-minute person-centred lifestyle conversation focused on diet and physical activity. An independent formative process and value-based evaluation was commissioned to examine the implementation of this programme in 14 primary care cluster areas across Wales during the initial roll-out.

**Methods:**

This evaluation was undertaken to ascertain the views on early implementation of the programme from service users, health care professionals and key stakeholders. The evaluation was informed by the Medical Research Council Framework for Process Evaluation and Wales Prudent Healthcare principles. As part of the value-based assessment, a preliminary cost-consequence analysis was conducted to understand the short-term economic impact of the implementation of the programme.

**Results:**

Service users who took part in the evaluation highly valued the programme and nearly half had been previously unaware that they were at risk of developing type 2 diabetes. Delivering the programme presented challenges but there was significant enthusiasm and support from all stakeholders. Overall, the programme was being delivered as intended albeit with evidence of some variation in the application of the programme eligibility criteria.

**Conclusions:**

In Wave 1 of the AWDPP roll-out, the intent to deliver the programme in line with Prudent Healthcare was successful and promising in terms of demonstrating value. Opinions expressed by service users suggest the AWDPP matters to them; raising awareness, promoting knowledge and capacity to change behaviours and motivate and raise confidence.

**Supplementary Information:**

The online version contains supplementary material available at 10.1186/s12889-024-19946-0.

## Background

Appropriate modification of the risk factors associated with type 2 diabetes, can prevent or delay development of the condition [1]. Studies that utilise lifestyle modification have demonstrated that the prevention of progression of type 2 diabetes can last for 10 years and longer [2]. Programmes to prevent diabetes, such as the ‘Healthier You Diabetes Prevention Programme’ provided by NHS England [3] have received global attention [4]. Whilst such programmes show promise in improving population health for people with prediabetes, implementation must take into account the local context and health system architecture. With a devolved UK health care system, evaluation of programmes aimed at improving population health in Wales requires comprehensive evaluation.

In Wales, 8% of the population are living with a diagnosis of diabetes [5]. The national diabetes prevention programme based on a brief lifestyle intervention was developed [6] following pilot work conducted in the Afan Valley and North Ceredigion primary care clusters [7]. The All Wales Diabetes Prevention Programme (AWDPP) began roll-out in April 2022, initially funded by Welsh Government across 14 Primary Care Cluster areas, two in each of the seven health boards in Wales. The intervention is based on the Transtheoretical Stages of Change model [8], and the COM-B implementation framework [9]. The intervention consists of systematic identification of adults at risk of developing type 2 diabetes (identified through glycated haemoglobin (HbA1c) measurement of between 42 and 47 mmol/mol [6 to 6.4%], i.e., prediabetes range) and offering a 30-minute person-centred lifestyle conversation (delivered face-to-face, by phone or by video call) focused on diet and physical activity. The intervention has been designed to be delivered by a designated Healthcare Support Worker (HCSW) who has received essential training to facilitate delivery of the AWDPP intervention, under the supervision of a dietitian [10]. Service users may then be referred on to existing health promotion / lifestyle modification programmes such as the National Exercise Referral Scheme (NERS) [11], weight management services e.g., Foodwise for Life, or the Let’s Prevent Diabetes interactive digital education programme [12].

To provide a comprehensive understanding of the implementation of the initial roll-out (Wave 1) of the AWDPP and to inform subsequent roll-out, Public Health Wales, who are responsible for delivering the programme in collaboration with local health board dietetic teams, commissioned a formative process and value-based evaluation to conceptualise the intervention context, understand the intervention delivery and implementation, examine the mechanisms of impact and to explore the value of the intervention, in line with Wales Prudent Healthcare Principles. The four principles of Prudent Healthcare are, (1) Achieve health and well-being with the public, patients and professionals as equal partners through co-production, (2) Care for those with the greatest health need first, making the most effective use of all skills and resources, (3) Do only what is needed, no more, no less; and do no harm, and (4) Reduce inappropriate variation using evidence-based practices consistently and transparently [13]. In this article we summarise the findings and insights of the full formative process and value-based evaluation [14].

## Evaluation methods

The interdisciplinary team conducting the evaluation comprised of a collaboration of researchers and public contributors from Swansea, Aberystwyth and Bangor Universities with wide ranging experience of diabetes (including diabetes education and prevention programmes), public health, health economics, qualitative research and programme evaluation. Two public contributors from the Diabetes Research Group Swansea Public Reference Panel were part of the evaluation team. The Public Reference Panel was established in 2015 to support the work of the Diabetes Research Unit Cymru, an all Wales organisation funded to support the development and delivery of diabetes research across Wales. Members of the panel were selected following an open call across Wales for volunteers, and represented people living with type 1, type 2 and prediabetes. Both public contributors working as part of the team for this evaluation were experienced in working with researchers in a lay capacity and experts through experience of prediabetes and type 2 diabetes. They were involved in the initial tender application, protocol development, evaluation questions and refining the evaluation methodology, including the development of participant materials and questions for surveys. They were also involved in interpreting and presenting the findings in an accessible final report. Members of the evaluation team have been involved in evaluating previous pilot diabetes prevention programmes conducted in Wales.

**Fig. 1:**
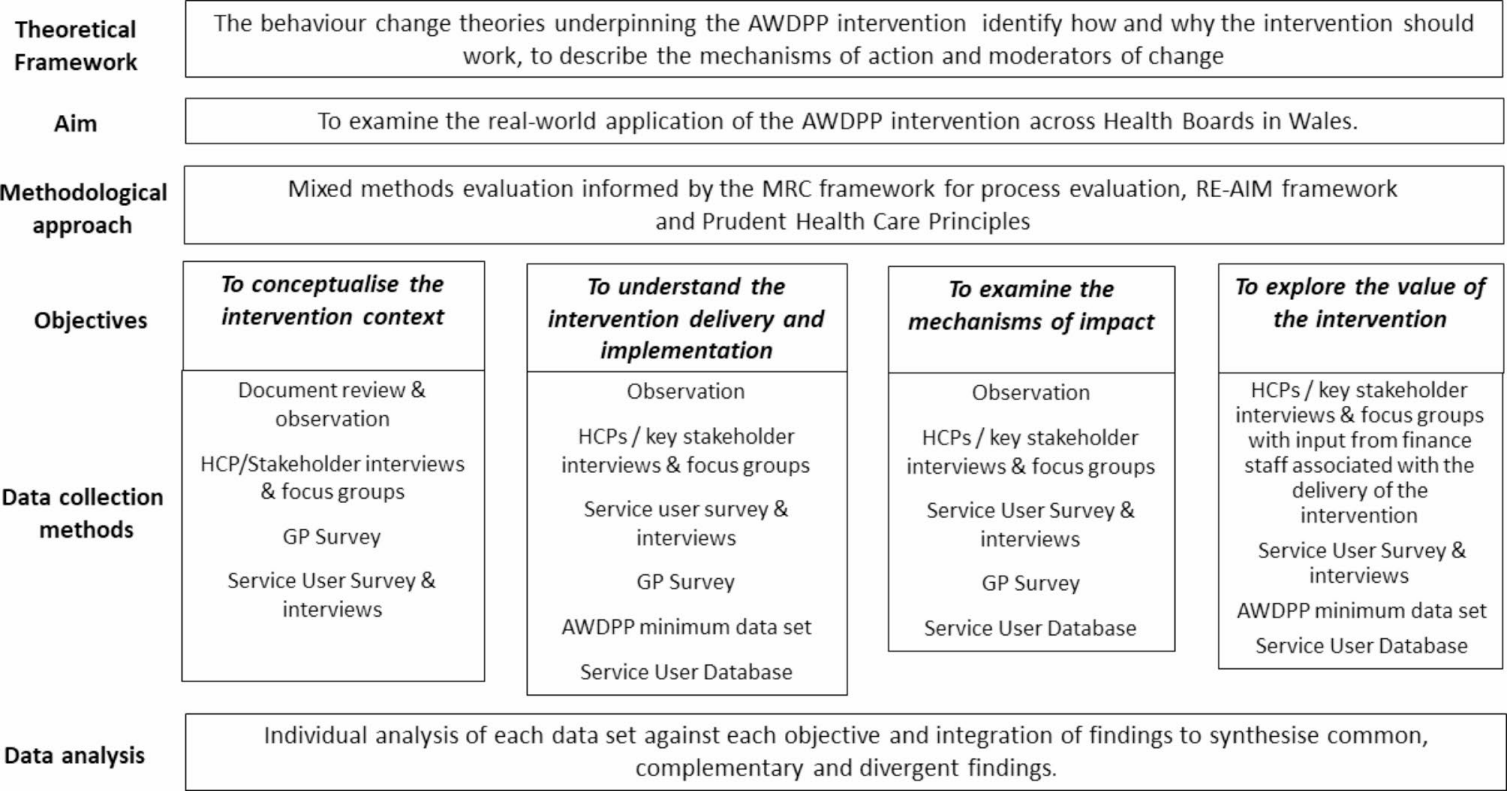
Evaluation Design

The formative process and value-based evaluation was undertaken between January 2022 and March 2023 to ascertain the views on the AWDPP intervention from service users, health care professionals and key stakeholders. The service users were aged 18 years and over and eligible for, or participating in, the AWDPP. The healthcare professionals were those involved in the development, implementation, delivery, management or support of the AWDPP (e.g., HCSWs, General Practitioners (GPs), practice managers and other general practice staff, diabetes and dietetic strategic leads). A mixed methods evaluation design was used that incorporated document review, interviews, focus groups, observations, surveys and analyses of anonymised routinely collected data (Fig. 1). The anonymised programme user data (AWDPP minimum dataset) was made available by Public Health Wales via the SAIL (Secure Anonymised Information Linkage) Databank [15] and from the AWDPP Service User Database collated by the programme delivery team (HCSWs and local dietetic leads).

### Overview of the AWDPP formative process and value-based evaluation design

The formative process evaluation covered the 14 Welsh Government funded primary care clusters across all seven Health Boards in Wales involved in Wave 1 implementation. Purposive sampling was used to ensure representation from communities across Wales, whilst maintaining achievable outcomes within the evaluation period.

The evaluation focused on measures of implementation, including reach, fidelity, adoption, and maintenance of the AWDPP [16], considering the context of evolving local healthcare environments, policies and priorities which may affect the successful implementation of a new model of care / health care promotion [17]. The guidance for process evaluations developed by the Medical Research Council (MRC) was followed [18, 19] and the evaluation was also informed by the Reach, Effectiveness, Adoption, Implementation and Maintenance (RE-AIM) Framework [20].

Data collection was obtained from: (1) document review, (2) observation of an AWDPP clinic using an observation checklist based on the template for intervention description and replication (TIDieR) framework [21], (3) service user survey, (4) individual interviews and/or focus groups with key stakeholders, health care professionals (HCPs) and service users, (5) analysis of the routine data collected via the AWDPP minimum dataset and Service User Database, and (6) GP survey. The data collection tools used can be found in Supplementary Information 1 to 6.

### Document review

The aim of the document reviews was to provide an understanding of the intervention context and the formal, organisational structures and processes, and provide a framework against which to compare and contrast findings from the evaluation data sources and perspectives.

A review was undertaken of mainly external documents to profile the AWDPP primary care setting at a cluster level for demographics, cluster population size, organisational characteristics, and the prevalence and burden of diabetes to understand contextual variation (Supplementary Information 7). This is important for designing, implementing, and evaluating effective diabetes prevention and management initiatives that are tailored to the needs of the local population. The profiling consisted of recent Welsh Government, Public Health Wales, health board and cluster documents that referred to diabetes prevention activity in Wales.

A review of internal AWDPP programme documents such as the intervention protocol [6, 10], was also completed to inform the evaluation design, develop our data collection tools (such as the interview and focus group topic guides, questionaries and observation checklist) and evaluate adherence to theory and specification to deliver the AWDPP. The documents were provided by the AWDPP team.

### Clinic observation

To assess the fidelity of the intervention to the AWDPP model and any modified local plans, a sample of the face-to-face and telephone/video clinic sessions were observed. Due to delays in programme start-up, clinics in only one primary care cluster area were established enough to undertake meaningful observations. One observer sat in on all service user appointments during a pre-arranged clinic session with the permission of the service users and HCSW. An observation checklist, based on the TIDieR framework [21], was completed, and field notes made of any variations to the original programme protocol. These variations were discussed with the HCSW delivering the intervention after the clinic had completed, to better understand the reasons behind it.

### Service user survey

Service user questionnaires were distributed by the local delivery teams to people attending AWDPP appointments between September and December 2022. The questionnaire consisted of 15 items and explored acceptability, accessibility, inclusivity and usefulness of the intervention, along with the reasons for agreeing to take part in the programme, and any facilitators and barriers to participation. The questionnaire was available in English and Welsh and could be completed in either a paper or online format. Service users were handed the paper questionnaire or a flyer giving the link to the online version at the end of face-to-face appointments, by the HCSW. Those who had a telephone or video appointment were sent the link to the online questionnaire via email, or the paper questionnaire after the appointment. A Freepost envelope was provided to those completing the paper questionnaire so that it could be returned directly to the evaluation team. Those who opted to complete the questionnaire online used a link or Quick Response (QR) code to access the questionnaire and submitted it online. All questionnaires were completed anonymously, however, at the end of the questionnaire, the service user had the opportunity to volunteer to take part in further research by giving their name and contact details. Demographic details of those who returned a service user questionnaire can be found in Supplementary Information 8.

### Interviews and focus groups

Service users who completed a questionnaire and expressed an interest to take part in further research were invited to take part in a focus group or one-to-one interview. No form of incentive was offered. The original design was to use purposive sampling to ensure representation from diverse settings with a wide range of circumstances that may influence responsiveness and accessibility to the AWDPP. However, due to the reduced evaluation timeframe caused by the longer than anticipated programme start-up, all 25 people who volunteered to take part in the additional research were contacted and invited to take part in either a one-to-one interview or focus group by phone or video call. By the end of the data collection period in December 2022, eight interviews had been conducted.

One-to-one semi-structured interviews and focus groups were also conducted via video call with key stakeholders including Lead Dietitians and HCSWs delivering the AWDPP, and national, local Health Board and Public Health Wales strategic leads. The data collection took place from September 2022 to January 2023. Topic guides were used to elucidate narrative data on their experience of the AWDPP. Discussions were recorded and transcribed verbatim.

### Routinely collected data

During the process of identifying and booking service users for the intervention, a Service User Database was populated by the healthcare professionals delivering the intervention to help them manage the booking process and record uptake of the programme. In addition to basic demographic data, any clinical reason for excluding potentially eligible service users, service user response to the invitation (including reason for decline) and any referral onto support services following the AWDPP appointment, such as weight management services, was recorded. Aggregated, anonymous data from the Service User Database was made available for the evaluation and provided an overview of how many people were going through the programme.

A minimum dataset containing anonymised service user level data such as basic demographic data, some clinical data and variables relating to the delivery of the programme was also analysed. These data were made available for the evaluation by Public Health Wales via the SAIL (Secure Anonymised Information Linkage) Databank [15], however in line with the guidance to use SAIL data, numbers less than 5 could not be reported. This minimum dataset was linked with other health records within SAIL such as the Welsh Index of Multiple Deprivation (WIMD).

### GP survey

All 88 General Practices within the 14 AWDPP funded cluster areas were invited to complete the GP online survey. The survey consisted of 15 items that measured the process of implementation from the perspectives of professionals directly and indirectly involved in implementing the AWDPP. The survey link was sent directly to each practice manager within the AWDPP funded clusters and could be shared with staff who were involved within the practice to allow multiple stakeholders the opportunity to complete and engage with the evaluation. The survey was open from November 2022 to the start of January 2023. An email reminder was sent to practices that had not responded after 1 month. A total of 25 individual responses were received.

### Assessing the value of the AWDPP programme

An exploratory cost-consequence analysis (CCA) was conducted to understand the short-term economic impact (technical value) of the implementation of the programme [22]. Using CCA is a recognised approach to assessing the value for money of a public health intervention and endorsed by the National Institute of Health and Care Excellence [23]. The perspective of the analysis was that of NHS Wales. The time horizon of the analysis available for the analysis was 6 months (June - December 2022). As this time horizon was less than 12 months, costs or outcomes were not discounted in line with best practice [23].

The resource use associated with the intervention was costed based on the AWDPP Intervention Protocol developed by Public Health Wales [10] and where there were gaps, information from the budget spreadsheet provided by the programme manager was used. Resource use was valued in pounds sterling (£) using the price year of 2022. Published unit costs (e.g., Personal Social Services Research Unit, NHS reference costs) were used to provide a UK-wide estimation. Where required, local finance records were used. Costs associated with the delivery of the AWDPP programme were estimated based on resource use and staff time required for training and contact with service users.

### Data analysis

The mixed methods evaluation was based on implementing quantitative and qualitative methods of data collection during the same timeframe and with equal weight placed on the findings derived from these sources. This approach broadly fits under the typology of triangulation where different data collection methods (as summarised in Fig. 1) are utilised. Analysis of quantitative data was primarily descriptive utilising mean, 95% confidence intervals, and percentages as appropriate. Statistical comparisons were limited due to differences in timelines and how the programme has been implemented across Health Boards. Analysis of qualitative data was largely inductive, drawing on the principles of thematic analysis [24]. Inductive themes were identified through examination and comparison, tabulation and mapping. Data were first analysed from each dataset and their results reported separately (e.g., survey). The evaluation team held weekly meetings throughout the evaluation period, in which emerging results were reported. The results were then brought together in the next stage of analysis and interpretation by integrating the data together against each of the objectives during the analysis and writing up stage. This activity was undertaken as part of several evaluation team meetings (each typically half day) to discuss and agree emerging findings, including meetings with public contributors. The focus was to integrate the findings to examine commonalities (e.g., where the results from each respective dataset were similar in explanation); complimentary findings (e.g., where the results from qualitative data added to or supplied more in-depth explanations from quantitative data) and divergence (e.g., where findings from one dataset were contradictory or deviant to the results from another on the same phenomenon of interest).

Ethical approval was obtained from NHS Wales Research Ethics Committee 3 (Reference:22/WA/0159) and all local Health Boards gave the necessary research permissions for the stakeholder and service user focus groups and interviews, survey distribution and clinic observations to take place. Interview and focus group participants gave written informed consent to take part in the evaluation.

## Results

A total of 187 people, including service users, key stakeholders and members of the local programme delivery teams, took part in the evaluation with representation across all local health boards. National clinical leads within the specialty of diabetes and representatives from the programme delivery team within Public Health Wales also took part, as can be seen in Table 1. To maintain anonymity, data from the qualitative interviews were labelled as HCP (for all participants in a professional role e.g., AWDPP team or GP staff) or service user. This is because the limited number of participants across professional role groups, Health Boards and service users could result in the potential attribution of quotes to individuals.

**Table 1:** Participation in the evaluation

	Stakeholder Focus Group	Stakeholder Interviews	HCSW Interviews	Service User Interviews	Service User Survey	GP Survey	Clinic Observations	Total
**Aneurin Bevan UHB**	1	2	2	3	20	3	0	**31**
**Betsi Cadwaladr UHB**	1	0	0	2	14	4	0	**21**
**Cardiff & Vale UHB**	3	1	2	2	35	3	6	**52**
**Cwm Taf Morgannwg UHB**	1	0	0	0	7	0	0	**8**
**Hywel Dda UHB**	1	2	2	0	0	1	0	**6**
**Powys THB**	1	0	2	0	18	4	0	**25**
**Swansea Bay UHB**	1	0	2	1	16	5	0	**25**
**Public Health Wales**	0	5	0	0	0	0	0	**5**
**National Strategic Lead**	0	3	0	0	0	0	0	**3**
**Anonymous**	0	0	0	0	6	5	0	**11**
***Total***	**9**	**13**	**10**	**8**	**116**	**25**	**6**	**187**

*The number of individual participants who took part in the qualitative data collection throughout the evaluation*,* broken down by local health board / organisation and data collection method.*

Service User data were collected between June and December 2022 during which time 3,158 people across 29 General Practices (11 of the 14 Primary Care Clusters initially funded) were deemed potentially eligible to take part in the programme. The key findings from the evaluation are shown in Table 2.

**Table 2:** Key findings from the evaluation

1. **Enthusiasm and Commitment**: Support for the AWDPP throughout the local Health Board teams was fully embedded in all roles and at all levels. Delivering the AWDPP across all seven Health Boards and in the initial 14 funded clusters presented implementation challenges, as would be expected when embedding a new national prevention programme into primary care. [Data sources: HCP interviews and focus groups, GP survey]
2. **Service User Participation in the AWDPP**: Between June to December 2022, 3,158 people were identified as at risk of developing type 2 diabetes and potentially eligible to take part in the programme across 29 GP practices. • During the evaluation period, 1,968 people were invited to take part in the programme. • The programme was deemed clinically inappropriate for 19% of people and they were not invited to take part. • At the end of December 2022, 1,015 people (52%) had accepted an appointment, 265 (13%) had declined and 688 (35%) had not responded to the invitation. • A total of 801 appointments were delivered either face-to-face or virtually and 68% of those who attended an appointment were referred to additional support services. *[Data source: Service user database]*
3. **Prediabetes Awareness**: Nearly half of those who attended an AWDPP appointment and completed the service user survey were unaware they were at risk of developing type 2 diabetes before receiving information about the programme. [*Data source: Service user survey and interviews]*
4. **Value to Service Users**: Our survey findings indicate that the AWDPP was highly valued by service users, providing an appointment tailored to their own personal needs and there is evidence that service users were willing to make lifestyle changes to address their risk factors. [*Data source: Service user survey and interviews]*
5. **Protocol Fidelity**: On the whole, the national protocol has been implemented as planned in the majority of Health Boards with one exception, where the Health Board had prior plans to implement a diabetes prevention programme Health Board wide and adopted the majority of the national protocol with some local modifications. Some exceptions are of note: • Evidence of some deviation from the original protocol with all Health Boards escalating service users over the age of 80 years for review by the dietitian. The agreed protocol was to escalate individuals over the age of 85 years for review. A large proportion of those escalated were being excluded from the programme. • Evidence that four Health Boards were escalating people with a BMI over 40 kg/m^2^ for dietetic review, and/or in some cases excluding them from the AWDPP. This may have been to refer individuals on to specialist weight management services rather than the AWDPP but there was some concern that weight management services were already busy and attending the AWDPP session might be helpful as a short-term intervention. • If appropriate, service users were referred onwards to the service that could best support their needs (e.g. National Exercise referral schemes or weight management services).The data suggested some variation in referral to support services being offered, e.g., those aged over 70 years had a lower referral rate than younger people, those living in the least deprived areas had the highest referral rate and women had a higher referral rate than men. These variations may have been related to local provision (e.g., availability of local services) or personal need / choice (e.g. tailoring to the service user. [*Data source: document review*,* clinic observation*,* service user database*,* service user survey and interviews*,* HCP interviews and focus groups]*
6. **Cost of AWDPP intervention**: Based on data up until December 2022, the estimated protocol based cost per AWDPP service user was £312. This includes the cost of follow up HbA1c and appointment at 12 months after initial appointment. As the programme continues to be rolled out and beds into practice it is probable that the cost per user will fall. [*Data source: document review*,* service user database]*

The primary enablers of the AWDPP implementation were felt to be the national protocol and national training programme. Data from the service users interviews and survey, and the HCP interviews and focus group confirmed that the training delivered enabled the HCSWs to gain useful knowledge, particularly around nutrition and improved their confidence and competence to deliver the AWDPP.

*“I think that the level of training that they did offer*,* especially the motivational interviewing*,* was really good quality. And I think just having those skills allows you to have a lot more broad conversations…”* [response from HCP interview].

Other enablers included the peer support of others implementing the national programme and the national Dietetic Lead role.

*“having the support network has probably been the most helpful thing with all of this”* [response from HCP focus group].

Good relationships and engagement with General Practices were also key to enabling implementation. From a service user perspective, those who attended an appointment and took part in the survey, found the time and location of the appointments on offer convenient and were motivated to attend by the desire to obtain further knowledge to reduce their risk of type 2 diabetes

“*I felt that there may be more I could learn about preventing or reducing my risk of diabetes”  [response from service user survey]*.

Data from the HCP interviews and focus groups suggest the barriers to implementation were primarily staff turnover and the grade of HCSWs assigned to the post.

*“The difficulty with this role is Band 3 levels within the NHS are normally stepping stones.”* [response from HCP interview].

*“…you’re then giving them a reasonably good amount of training*,* and then there’s other job opportunities at a higher grade… there’s going to be quite constant movement with the people that are out there on the ground.”* [response from HCP interview].

Recruitment of HCSWs was time consuming in most of the Health Board areas so regular staff turnover, in small teams with little or no cross cover, directly impacted the delivery of the programme. Further barriers were insufficient training in IT systems used for accessing primary care held information and a lack of central support for this and negotiation and sign-off of data sharing agreements.

Some service users, when interviewed, felt that not knowing what to expect from the programme may have been a possible barrier for those who had not engaged, and it could have been made clearer in the service user information that this was a diabetes prevention programme, rather than weight management programme which was seen as a deterrent to some service users.

*“I can see how some people would think*,* oh I’m not going to that*,* I don’t need them to tell me that I’m overweight and that I should be exercising*, I know it” [response from service user interview].

Data from the Service User Database collected by the local delivery teams indicated that a very small number of people who responded to the invitation letter declined an AWDPP appointment because of work or caring responsibilities (14/265, 5%). Some flexibility in appointment time may have accommodated these issues but it was not highlighted as a major barrier for those who took part in the evaluation. The most common reason for not taking up the offer of an appointment was because they felt they already had enough information (71/265, 27%).

It was also felt the programme could be made a little more inclusive by actively inviting a partner or other family member to attend the appointment who could provide support, not only during the appointment but also with making and maintaining lifestyle changes. The point was made that people often share or rely on others to provide food so dietary changes can impact others if part of a household.

Whilst each Health Board was provided with central programme funding for 0.25 Full Time Equivalent (FTE) Lead Dietitian per primary care cluster, much more time was being spent by the Lead Dietitians in all Health Boards during Wave 1 of the AWDPP implementation. This was partially because a large proportion of potential service users were escalated to the Lead Dietitian for review, and subsequently assessed to be clinically inappropriate for the programme. Learning from this, the reasons for escalation and subsequent exclusion from the programme need to be understood as there may be a case for reviewing the inclusion / exclusion criteria and revising the search template used to identify people for the programme in GP practices.

For Wave 1, based on the activity up to December 2022 in the 29 practices that had taken part across the 7 health boards, the AWDPP cost £218,225 to NHS Wales, with an estimated all-inclusive cost to enable one person to attend an AWDPP appointment of £272. As the AWDPP protocol requires service users to receive a follow-up HbA1c and an appointment at 12-months post-intervention, inclusion of these costs into the estimates increased the NHS Wales total cost to £249,978, with an estimated cost per session delivered in Wave 1 of the roll-out of the AWDPP of £312.

## Discussion

In the roll-out of Wave 1 of the AWDPP, the intent to provide and deliver the programme in line with the principles of Prudent Healthcare was successful and showed potential in delivering against all four principles as the programme matures. Prudent Healthcare puts people at the centre of decisions about their own health. Instead of clinicians making all the decisions about treatment, these are shared decisions between practitioner and patient – this is an important part of co-production.

The AWDPP is an exemplar of the first principle of Prudent Healthcare, giving people receiving the brief intervention and related support (e.g. referral to other follow on services such as NERS), the knowledge and power to address their high blood glucose and the reasons for changing their lifestyle to reduce their risk of developing type 2 diabetes and improve their health and well-being. The second principle of Prudent Healthcare encourages the most effective use of skills and resources. By providing the AWDPP intervention at an early stage in the pathway of diabetes care, considerable NHS resources can be released in the future, potentially saving millions of pounds by avoiding considerable morbidity and mortality associated with diabetes. GPs and dietitians are highly trained and scarce resource: in the context of the AWDPP training up the HCSW to undertake a brief intervention, support people who participate by co-producing their plan for addressing the prediabetes, plus signposting the person to other resources that are relevant and useful, is in perfect alignment with Prudent Healthcare. When ‘more’ is needed then the pathway is there to escalate referrals to a dietitian or GP.

By providing the AWDPP brief intervention at an early stage in the pathway to diabetes, the third principle is supported by doing only what is needed, no more no less. However, in trying to optimise the contact between the HCSW and the person who is at risk of type 2 diabetes there may be a tendency to try and do too much in the 30 minute appointment. The fourth principle, reducing inappropriate variation using evidence-based practices consistently and transparently is upheld by rolling out a programme across all health boards in Wales, albeit with some health boards taking different approaches to the roll-out.

Drawing upon our findings the key lessons learnt from our evaluation are shown in Table 3.

**Table 3:** Key lessons learnt from the formative process and value-based evaluation

1.** Buying in to the Value of AWDPP**: A consistent and powerful message from stakeholders, HCPs and service users was the potential value they felt the AWDPP had in reducing health problems related to diabetes and the burden of diabetes on the NHS.
2. **Ambition vs. Reality**: The implementation of the AWDPP was an ambitious programme of work and required complex processes, planning, engaging and delivery to be adopted, at pace and at some scale, across the 14 Wave 1 primary care clusters across NHS Wales Health Boards. Due to this complexity, the ambitions of the AWDPP timeline were occasionally inconsistent with the realities and pace of working on the ground in the Health Boards.
3. **National vs. Local**: There is an important trade-off between implementing a national ‘protocol-driven’ programme of change versus the realities of how this is implemented for local context and needs. The challenges perhaps seen in this phase of the national roll-out may have been due to this being a ‘top-down’ approach rather than the ‘bottom-up’ approach used within the pilot work. Our evidence suggests that central funding and local engagement were central to the governance and management of the AWDPP during this early phase.
4. **Operationalising Theory of Change**: Our findings suggest there are some gaps in how the theory of change has been operationalised in practice and its use could have been more explicit throughout the roll-out e.g., to check that those on the ground understood the theory of change underpinning the AWDPP in order to apply it in practice. A potential area to consider as the AWDPP moves towards looking at the outcomes from attending the programme, is how the programme fits within other prevention programmes such as weight management in ensuring it is specifically addressing and can demonstrate how it is preventing type 2 diabetes.
5. **Local Capacity**: Related to the above, our evaluation was too ‘early stage’ to fully assess but the capacity to manage consequences and actions e.g., referrals onwards to NERS and other follow-up programmes, was variable. This is likely to be an important part of moving the AWDPP towards translating into outcomes as part of a pathway of preventative care.
6. **Equity of Access**: At the time of this evaluation, it was premature to determine whether or not there was equitable access to the AWDPP, and related to this, understanding why eligible service users did not take up the intervention. Our findings to date suggest that clearer messaging around type 2 diabetes prevention rather than general weight management may encourage engagement with the programme. Further exploration could be undertaken to understand the reasons why eligible service users, particularly those with a higher BMI and from areas of highest deprivation did not take up the AWDPP intervention.
7. **Pace vs. Practicality**: Managing the grant allocated to the Health Boards and the practicalities of managing the funding and tasks caused a few issues at Health Board level at the beginning of the AWDPP roll-out, which have been resolved. Whilst acknowledging the pace of the roll-out that occurred was necessary, identifying key contacts, establishing relationships and the communications between the local teams managing the budget and the central teams can be enhanced as further waves of roll-out occur. Stakeholder interviewees highlighted the need for good relationships with local teams when managing such a big programme and needing to keep to timelines.
8. **Recruitment and Retention**: In all Health Boards, as the AWDPP was rolled out financial management and ability to use the budget available was affected by the challenges of recruiting and retaining the Band 3 HCSWs, resulting in slippage and underspend to a greater or lesser extent. Planning the next rollouts with a longer timeline and investigating ways of managing slippage because of staff recruitment processes will enable more effective use of available budget.
9. **Roles and Responsibilities**: Our findings suggest that roles and responsibilities were critical components to AWDPP. Despite core funding in place there were variances across Health Boards e.g., additional resources given to dietitian support beyond the core allocation in some areas. There were also differences in titles, banding and contracts for HCSWs, which may in part explain the challenges identified in recruiting and retaining staff. Opinion varied as to whether the HCSW role was a Band 3 or Band 4 but were unanimous in their concerns that a fixed term Band 3 HCSW (as originally budgeted) to deliver the role was not conducive to sustainability of the programme.
10. **IT and System Access**: The system context of delivering the AWDPP is critical. IT and GP system access hampered progress in the roll-out and delivery of the AWDPP, and these were evidenced at national and local level. Investment in IT training to use GP software for the AWDPP delivery workforce was inconsistent. Going forward, it was felt that access to GP systems should be agreed at the beginning of the programme at a cluster level rather than negotiating at an individual practice level.

### Key lessons learnt from the formative process and value-based evaluation

There was significant enthusiasm and support for the AWDPP throughout the Health Board teams in all roles and at all levels. However, some local practicalities of the roll-out absorbed much time and resource putting the local timelines back, in some cases significantly. Individual Health Boards took different approaches to rolling out the programme and, in some places, establishing the role, banding and job description for the AWDPP HCSW introduced variation of delivery and the pace of delivery differed.

Our survey findings indicated that the AWDPP intervention was highly valued by service users in providing an appointment tailored to their own personal needs, with a high rate of attendance and evidence from the qualitative research that service users were willing to make changes in their lifestyle to address their risk factors.

As part of learning lessons from the AWDPP evaluation to date, our methods and findings have been considered in context to the work reported on the English and Scottish Diabetes Prevention Programmes (DPP). The English evaluation was organised under the broad umbrella of the DIPLOMA evaluation which was funded by the National Institute for Health and Care Research (NIHR) via a commissioned HS&DR call with £2,790,952 awarded over 5 years (April 2017-April 2023). Our evaluation was separately commissioned and covered a significantly shorter timeframe.

England’s NHS DPP has reported several papers of its evaluations set out in 8 work packages, and a narrative review of the findings and lessons from the DIPLOMA evaluation has also been reported [25]. To enable a reasonable ‘like for like’ comparison with the AWDPP programme at its current time horizon, we have focused on those papers reporting the early-stage findings that are generally aligned to the objectives we set out in our evaluation protocol.

The early lessons of implementing the NHS DPP were reported by Stokes et al., in 2019 [26]. This work focused on informing the sampling strategy to inform the selection of case sites as part of the wider, longitudinal DIPLOMA evaluation. Whilst this was not an explicit strategy of our formative process and value-based evaluation, our findings around the context and mechanisms of impact have indicated that there are several factors that align to the DIPLOMA evaluation team’s findings. Their first three key findings resonate with ours in terms of (1) managing new providers, (2) promoting awareness, and (3) recruiting service users. It was difficult to compare to the English funding model, however, as shown in our findings from the value assessment, there are important issues regarding funding and sustainability of the AWDPP to consider.

A recent paper has suggested that the introduction of the NHS DPP in England reinforces existing inequalities in care, with those registered in primary care practices that provide lower quality clinical care becoming even more disadvantaged [27]. We did not identify any such inequalities or unintended consequences other than evidence suggesting that some service users expressed other health concerns (e.g., mental health) but there were limited avenues within the AWDPP for signposting other than to the GP. We suggest that such unintended consequences should be examined in any future evaluation of the AWDPP.

To explore equity considerations further there are potential benefits of the AWDPP team working closely with the SAIL databank to provide opportunities to understand how the AWDPP intervention can change outcomes in the Welsh population, and we suggest that capturing data to formally inform equity considerations is a priority for future evaluations. Our evaluation also showed the potential to include capturing patient reported outcomes such as Health Related Quality of Life (HRQOL) using the EQ-5D 5 L questionnaire to add value to how we understand the difference and impact that a diabetes prevention programme makes to people and populations.

The findings from DIPLOMA from several investigations of the fidelity of the NHS DPP showed wide variation and fidelity drift across participants [28] and providers [29] and suggest that fidelity must be thoroughly considered as part of any future summative process evaluation. Our limited assessment found that, on the whole, the protocol was being delivered as planned with some variation across the majority of health boards in how the eligibility criteria were being applied. This is with the exception of one health board that had already taken the decision to implement a diabetes prevention programme health board wide, based on the brief intervention model, prior to the AWDPP being funded.

Scotland has also recently reported on their qualitative process evaluation of their Framework for the Prevention, Early Detection and Intervention of Type 2 Diabetes [30]. This focused on the implementation in three early adopter sites. Whilst there are clear differences in how the programme was implemented compared to AWDPP (e.g., digitalisation of the intervention as part of the COVID-19 challenges), there are common findings between our two evaluations. The motivations for service users in Scotland to join were around concern about prediabetes, desire to improve health or recommendation by a professional. They also found that service users were positive about their experience and changes made as a result of participation. Consistent with our findings, information governance was a barrier, alongside concerns about sustainability of funding beyond the current allocation. The importance of key staff was pivotal to their success, and similarly the early sites found challenges in staff retention.

Navigating relationships was challenging but, similar to our findings, enabled many lessons to be learnt to strengthen interactions and collaborations across different teams and regions. In line with our findings, the engagement of primary care staff was a key determinant of success. Their conclusions focused on recommendations around (1) wider range of programme options; (2) financial support and commitment to longer-term planning; (3) partnership working and feeding the findings from these early adopters into wider roll-out to ensure shared vision and common understanding (4) improved systems around information governance and (5) building relationships with primary care.

### Strengths and limitations

We successfully applied a robust evaluation framework to deliver early findings and lessons. Another strength of our evaluation was the inclusion of a wide range of stakeholders, delivery staff and service users in order to make sure all aspects of the Wave 1 AWDPP implementation was represented. This enabled the evaluation to produce robust, real-world, and timely evidence on the challenges and to work on solutions with the implementation team to guide the next wave of the roll-out and subsequent evaluation. One Health Board was an early adopter of the programme and quick to establish clinics in one of their primary care cluster areas. This Health Board is therefore represented more than others in the evaluation sample and was the only area available during the data collection period with established clinics that could be observed. We were limited by the number of participants who took part in the AWDPP during the data collection period, and the number of HCSWs that could contribute to the interviews and focus groups because of delays in staff recruitment and local programme start-ups. Because of the later than anticipated start, the time available to carry out the evaluation was reduced. We also acknowledge the potential for bias in our reporting as we focused on those participating in the AWDPP rather than those who chose not to or could not engage with the programme. Due to the lack of available data, we were unable to analyse differences in how the AWDPP has been delivered across ethnicities and across disability status. We were therefore unable to make any comments on these equity concerns. These limitations, however, are unlikely to impact research credibility. Whilst the scope and direction of future evaluations will be led by the AWDPP team, our findings may help future teams in mitigating the limitations we encountered and enable the current logic model to be adapted to further understand how the AWDPP is making an impact on preventing type 2 diabetes (e.g. longer-term outcomes captured).

## Conclusion

In conclusion, after approximately one year of the roll-out of AWDPP (Wave 1) the intent to provide and deliver the programme in line with Prudent Healthcare was seen to be successful, delivering against all four principles, and is promising in terms of demonstrating value. Inevitably, as this is an ambitious and complex programme to roll-out in a short period of time, there were some challenges. None of these are unsolvable and indeed the stakeholder community interviewed have proffered solutions. We have offered points for consideration and our findings can be used to inform the subsequent phases of the AWDPP roll-out across Wales and inform the growing global evidence base of the implementation of diabetes prevention programmes.

## Electronic supplementary material

Below is the link to the electronic supplementary material.

Supplementary Material 1. Observation Checklistpdf fileObservation of AWDPP DeliveryObservation checklist.

Supplementary Material 2. Service User Interview and Focus Group Topic Guidepdf fileService user interviews and focus groupTopic guide.

Supplementary Material 3. Stakeholder Interview and Focus Group Guidepdf fileStakeholder Interview and Focus GroupsTopic guide.

Supplementary Material 4. HCSW interview guidepdf fileHCSW interview guideTopic guide.

Supplementary Material 5. Service User Questionnairepdf filePatient QuestionnairePatient questionnaire.

Supplementary Material 6. General Practice Questionnairepdf fileGeneral Practice QuestionnaireGeneral Practice questionnaire.

Supplementary Material 7. Primary Care Cluster Demographicspdf filePrimary Care Cluster DemographicsTable of primary care cluster area demographic details.

Supplementary Material 8. Service User Survey Demographicspdf fileService User Survey DemographicsTable of service users demographic details.

## Data Availability

The datasets used and/or analysed during the current study are available from the corresponding author on reasonable request.

## Abbreviations

AWDPP: All Wales Diabetes Prevention Programme
CCA: Cost consequence analysis
DPP: Diabetes Prevention Programme
EQ-5D 5L: EuroQoL 5 Dimension 5 Level Questionnaire
FTE: Full time equivalent
GP: General Practitioner
HbA1c: Glycated haemoglobin
HCSW: Healthcare Support Worker
HCPs: Health Care Professionals
HRQoL: Health Related Quality of Life
IT: Information Technology
MRC: Medical Research Council
NERS: National Exercise Referral Scheme
NHS: National Health Service
RE-AIM: Reach, Effectiveness, Adoption, Implementation and Maintenance Framework
TIDieR: Template for intervention description and replication
SAIL: Secure Anonymised Information Linkage
UHB: University Health Board
THB: Teaching Health Board
