# Supplementary material for: The challenges and lessons from a formative process and value-based evaluation of the wave 1 roll-out of the all Wales Diabetes Prevention Programme
Source: BMC Public Health. 2024 Sep 13;24:2499. doi: 10.1186/s12889-024-19946-0 (PMC11401378; doi:10.1186/s12889-024-19946-0)
Supplement: Supplementary file 1 — Supplementary Material 1. Observation Checklistpdf fileObservation of AWDPP DeliveryObservation checklist. [file 12889_2024_19946_MOESM1_ESM.pdf]

Observation of AWDPP Delivery

SABU Observer	
Date of observation	
HCSW delivering AWDPP intervention	
GP cluster / Health board	
**Session start time**	
**Session end time**	

Data	Comments
**Mode of delivery**<br>An individual face-to-face basis, virtual via telephone or video consultation (including if individual or group session)	
**Location** (if F2F)<br>• Type of venue (e.g., GP surgery, community centre)<br>• Venue/ room details (describe the room in terms of accessibility, size, equipment)	
**HCSW delivering session**<br>• Length of time delivering AWDPP<br>• Length of time since training<br>• Professional background<br>• Sex	
**Service user**<br>• Sex (M/F)<br>• Support persons present (e.g., family, carers)	
**Aims & objectives of AWDPP / current session explained to patient**	
**Materials** (patient resources)<br>Describe physical or informational materials given out or used in delivery<br><br>Do annotate if the information was shared as physical paper version (Yes - P) or if links to electronic copies were shared (Yes - E).	<table><tr><th>Leaflet</th><th>Yes, received (P/E)</th><th>No, not received</th></tr><tr><td>Eatwell Guide</td><td></td><td></td></tr><tr><td>Diabetes UK “Eating Well”</td><td></td><td></td></tr><tr><td>Diabetes UK “Be Active”</td><td></td><td></td></tr></table><br>**Any others – describe:**
**Tailoring (**Planned adaptions)<br>Describe personalisation or adaptation (what, why, when, how) e.g., signposting / referrals to Foodwise for Life / the National Exercise Referral Scheme / other local services	

**Modifications** (Unplanned alterations)<br>Describe changes to planned intervention (what, why, when, how)	
**Fidelity**<br>Describe the extent to which the intervention was delivered as planned.	
**Rapport**<br>Describe to what extent there is collaboration and parity between patient and HCSW.	
**Goal setting**<br>Describe to what extent the goals are person centred and if the patient is given autonomy to agree own goals (e.g., is the HCSW supporting the conversation and providing examples where appropriate which may contribute to achieving goal success)	
**Any other observations / comments** (e.g., age, disability, acceptability of intervention accessibility of presentation and location)	
