# Supplementary material for: The challenges and lessons from a formative process and value-based evaluation of the wave 1 roll-out of the all Wales Diabetes Prevention Programme
Source: BMC Public Health. 2024 Sep 13;24:2499. doi: 10.1186/s12889-024-19946-0 (PMC11401378; doi:10.1186/s12889-024-19946-0)
Supplement: Supplementary file 2 — Supplementary Material 2. Service User Interview and Focus Group Topic Guidepdf fileService user interviews and focus groupTopic guide. [file 12889_2024_19946_MOESM2_ESM.pdf]

Service User Interviews and Focus Groups

The key topics for the interviews will be around exploring the patients experiences and perceptions of the AWDPP delivery including accessibility and acceptability.
We will explore the patients':

- perceptions of the target group inclusion / exclusion and acceptability of AWDPP (reach, i.e., ***Who is taking part in the intervention?***)
- consideration of what matters to the patients and how patients are involved in decision making (value-based health care / effectiveness, i.e., ***Value based care / is the work in line with prudent healthcare principles?***)
- rapport with and perception of the HCSW and their skills (adoption, i.e., ***Who is delivering the intervention?***)
- experience of the AWDPP (implementation, i.e., ***Is the intervention being implemented as planned? What aspects of the programme are working well and not so well? What are the enablers and barriers to implementation?***)
- perception of their motivation and capability to engage with (maintain) the behaviours identified in the intervention (maintenance / sustainability, i.e., ***How can any improvements be sustained?***)

The questions below can be asked in any order depending on how your conversation goes and all questions do not have to be asked, nor in the way presented here – you can use your own words and some interviewees will not need many /any prompts.

For focus groups, emphasis on access, acceptability, and barriers / facilitators for participation in programme (both in terms of attendance of session and proposed activities for prevention).

1-99 = Question topics we are interested in

a-z = Prompts related to the topic that you can use to expand on the question or help get the interviewee talking if they don't really have much to say

1. **Can you tell me about your invitation to the AWDPP?**
    a. *What information in the invitation letter was attractive / made you make an appointment?*
    b. *What impact did it have on you to be told you were at risk of developing diabetes?*

2. **What did you expect / wish from a diabetes prevention programme?**

3. **Tell me about your experience of the AWDPP session.**

4. **How convenient was it for you to attend the session?**
    a. *Ease of access to venue / time/ frequency of sessions*

5. **How did you find the resources or information suggested/provided for you?**
    a. *Any consideration of/issues with education levels, financial issues, gender issues or cultural beliefs?*

6. **How easy / difficult did you find it to set goals?**
    a. *What role did the HCSW take in helping you with your goals development?*

b. *Do you think the referral options are appropriate?*

7. **How well are necessary resources readily available to make and maintain lifestyle changes?**
    a. *access to shopping / cooking facilities, exercise, time to pursue healthy activities*

8. **How motivated (confident) are you in doing physical activity /managing your weight / healthy eating?**
    a. *What would support you to stay motivated / become more confident?*
    b. *How supportive are friends and family?*

9. **What facilitated your attendance?**
        i. *personal experience of a friend or family, convenience, incentives*
    b. *What were barriers?*
        i. *time, cost, location*

10. **Are there any (social and cultural) barriers to you attending the programme?**
    a. *Are there any solutions to the barriers?*
    b. *How could we get more people to participant in an intervention like this?*

11. **How could we get more people to participant in an intervention like this?**

12. **Were you referred for further support?**

13. **What is your perception on the knowledge / interest / skills of the HCSW delivering AWDPP session?**
    a. *What is your perception on the characteristics of the HCSW delivering the intervention?*

14. **What could be added to the programme?**
    a. *Would you want to gain more information?*

15. **Is there anything else you would like to share with the process evaluation team at this time?**
