# Supplementary material for: The challenges and lessons from a formative process and value-based evaluation of the wave 1 roll-out of the all Wales Diabetes Prevention Programme
Source: BMC Public Health. 2024 Sep 13;24:2499. doi: 10.1186/s12889-024-19946-0 (PMC11401378; doi:10.1186/s12889-024-19946-0)
Supplement: Supplementary file 3 — Supplementary Material 3. Stakeholder Interview and Focus Group Guidepdf fileStakeholder Interview and Focus GroupsTopic guide. [file 12889_2024_19946_MOESM3_ESM.pdf]

Stakeholder / Strategic Lead Interviews and Focus Groups

Interviews undertaken with local stakeholders and delivery teams will ascertain details of the planned programme, along with any local modifications at the beginning of the evaluation, e.g.:

- decisions around the target group inclusion / exclusion by the strategic leads (reach, i.e., ***Who is taking part in the intervention?)***
- considerations of value-based care and prudent health care principles related to the AWDPP delivery (value-based health care / effectiveness, i.e., ***Value based care / is the work in line with prudent healthcare principles?)***
- the staff recruitment process for delivery of the AWDPP (adoption, i.e., ***Who is delivering the intervention?***)
- experience of the AWDPP implementation (***Is the intervention being implemented as planned? What aspects of the programme are working well and not so well? What are the enablers and barriers to implementation?)***
- continued support of the AWDPP and how the programme can be made sustainable (maintenance, i.e., ***How can any improvements be sustained?)***

The questions below can be asked in any order depending on how your conversation goes and all questions do not have to be asked, nor in the way presented here – you can use your own words and some interviewees will not need many /any prompts.

For focus groups, emphasis on programme development, stakeholder inclusion, adoption, acceptability and barriers / facilitators for delivery.

1-99 = Question topics we are interested in

a-z = Prompts related to the topic that you can use to expand on the question or help get the interviewee talking if they don't really have much to say

1. **Tell me about your role and extent of involvement with the AWDPP.**
2. **What did you expect / wish from a diabetes prevention programme?**
3. **What resources have you committed to this…**
4. **What do you see as the key aims of the AWDPP?**
    a. *What could be added to the programme?*
5. **What are the key elements of the initiative that must be delivered to be successful?**
6. **Who will benefit from the AWDPP?**
    a. *Primary /secondary care*
7. **Can you describe the degree to which the recruitment rate of participants met your expectations?**
    a. *Do you think the inclusion criteria is accurate? Inclusive enough?*
    b. *Where the searches appropriate / did they flag up the correct patients?*

c. *What strategies do you think worked best to recruit the target audience?*
d. *Could anything have been / be done differently to reach more people?*

8. **What is the expertise or characteristics of those you are targeting to deliver the AWDPP?**
    a. *Do any characteristics differ from the targeted recruitment and those who deliver the AWDPP (e.g., education, training, capacity)?*

9. **Can you describe the degree to which the recruitment rate of staff met your expectations?**
    a. *What strategies do you think worked best to recruit staff members?*
    b. *What could the team have done better to reach more staff members?*
    c. *What are your perceptions of training that was offered for HCSW/Staff (Duration, frequency, content)?*
    d. *Has training been delivered as planned?*

10. **Has AWDPP been implemented according to plan?**
    a. *What implementation challenges have been identified and how are they overcome?*
    b. *What adaptations or modifications do you think are necessary to help implement the AWDPP?*
    c. *How do you know what adaptations or modifications were made locally to the AWDPP?*
    d. *What quality assurance steps are taken by AWDPP?*

11. **What would have happened if there was no government funding?**
    a. *To what extent would some/none/all of it have happened without the funding?*

12. **What will help or hinder the AWDPP to achieve its objectives and outcomes?**

13. **Any gaps/areas for improvement?**

14. **How can the Programme be made sustainable?**
    a. *Any elements already sustainable/embedded/funded by elsewhere?*
    b. *Can GP clusters / HB's sustain the initiative over time and are there plans to leave trained staff in place?*
    c. *What infrastructure will be needed to sustain the initiative?*
    d. *Is there an infrastructure and funding that will remain?*

15. **Is there anything else you would like to share with the process evaluation team at this time?**
