# Supplementary material for: The challenges and lessons from a formative process and value-based evaluation of the wave 1 roll-out of the all Wales Diabetes Prevention Programme
Source: BMC Public Health. 2024 Sep 13;24:2499. doi: 10.1186/s12889-024-19946-0 (PMC11401378; doi:10.1186/s12889-024-19946-0)
Supplement: Supplementary file 4 — Supplementary Material 4. HCSW interview guidepdf fileHCSW interview guideTopic guide. [file 12889_2024_19946_MOESM4_ESM.pdf]

Health Care Support Worker Interview Guide

The key topics of the HCSW interviews will be how the programme is being implemented, barriers and enablers for successful implementation and any recommendations for the further development of the programme and all Wales implementations, e.g.:

- perceptions of the target group inclusion / exclusion and acceptability of AWDPP (reach, i.e., ***Who is taking part in the intervention?)***
- consideration of what matters to the patients and how patients are involved in decision making (value-based health care / effectiveness, i.e., ***Value based care / is the work in line with prudent healthcare principles?)***
- role, skill set, motivation, and capability to deliver the AWDPP (adoption, i.e., ***Who is delivering the intervention?***)
- experience of delivering the AWDPP (implementation, ***Is the intervention being implemented as planned? What aspects of the programme are working well and not so well? What are the enablers and barriers to implementation?***)
- perception for continued delivery of the AWDPP and future roll out (maintenance / sustainability, i.e., ***How can any improvements be sustained?***)

The questions below can be asked in any order depending on how your conversation goes and all questions do not have to be asked, nor in the way presented here – you can use your own words and some interviewees will not need many /any prompts.

The 'flow' of questions here follows the process that we first ask about the HCSW's role and training to get them talking about something known to them, i.e., their experience, and then move to the programme and questions about the patients.

1-99 = Question topics we are interested in

a-z = Prompts related to the topic that you can use to expand on the question or help get the interviewee talking if they don't really have much to say

1. **Can you tell me a bit about what motivated you to apply for this post?**

2. **What do you need to know to deliver this programme?**
   a. *What training did you receive?*
   b. *Was the training you received enough?*
   c. *what did you learn?*
   d. *Did the training include watching other providers?*
   e. *Describe how well you feel equipped to deliver the program based on the training you received?*

3. **How confident are you in your skill and capability to deliver this programme?**
   a. *Are you more confident in some areas (activities) than others?*
   b. *What are the challenges in your work?*

4. **What do you hope to achieve by delivering the programme?**
   a. *How rewarding is delivering this programme?*
   b. *Do you receive any feedback?*
   c. *What is the best thing about your job?*

5. **How important do you think diabetes prevention is in general?**

   a. *Why do you think it is / isn't important?*

6. **Do you think the AWDPP inclusion criteria is accurate?**
   a. *Inclusive enough?*
   b. *Where the searches appropriate / did they flag up the correct patients?*
   c. *Do you think the way you were identifying patients is acceptable for the patients themselves? Why/why not/for whom?*

7. **Are there any barriers to people attending the AWDPP?**
   a. *Are there any solutions to the barriers?*
   b. *Is there anything that helps / can help people attending?*

8. **Tell me about a typical AWDPP session.**
   a. *To what extent has the sessions needed to be modified?*
   b. *Why? Describe process for modifying the plan (how need for modification was discovered; how did this change impact timeline, design, or other factors)*
   c. *How do you report what adaptations you have to make in the field?*
   d. *How do you know the adaptation is needed?*
   e. *How do you report fidelity to intervention delivery?*

9. **What is needed to deliver the AWDPP session?**
   a. *Equipment – e.g., Laptop/ pc / phones / internet*
   b. *Venue*
   c. *Systems / access*
   d. *Other?*
   e. *Do you have access to what you need?*

10. **How convenient is the delivery format / venue for you?**
   a. *Anything else important about this format / venue for you?*

11. **Is there an opportunity to explore what matters to the patients?**
   a. *(How) are patients involved in decision making?*

12. **How did you find the process of referring patients?**
   a. *Do you think the way you are referring patients is acceptable for the patients themselves? Why/why not/for whom?*

13. **What aspects of the programme are working well and not so well?**
   a. *What are the enablers and barriers to implementation?*
   b. *Any gaps/areas for improvement?*

14. **What would be needed for other staff to deliver the intervention in the future?**

15. ***Is there anything else you would like to share with the process evaluation team at this time?***
