# Supplementary material for: The challenges and lessons from a formative process and value-based evaluation of the wave 1 roll-out of the all Wales Diabetes Prevention Programme
Source: BMC Public Health. 2024 Sep 13;24:2499. doi: 10.1186/s12889-024-19946-0 (PMC11401378; doi:10.1186/s12889-024-19946-0)
Supplement: Supplementary file 5 — Supplementary Material 5. Service User Questionnairepdf filePatient QuestionnairePatient questionnaire. [file 12889_2024_19946_MOESM5_ESM.pdf]

All Wales Diabetes Prevention Programme

Patient Questionnaire

We would really like to hear about your experience of attending the All Wales Diabetes Prevention Programme (AWDPP).

This short programme has been developed to help people make changes which might prevent or delay the onset of Type 2 diabetes. It has been designed especially for people who are at risk of developing Type 2 diabetes. The programme is new and we want to know how well it works and how it can be improved to help other people at risk.

We are a group of researchers from Swansea, Aberystwyth and Bangor Universities and have been commissioned by Public Health Wales to find out how you feel about the programme, how easy or difficult it was to take part and how useful it was to you.

Your views will help healthcare professionals design better and more effective programmes in the future. Please complete this form. It will take approximately 10 minutes and then return in the FREEPOST envelope provided. Alternatively you can complete the questionnaire online by typing this link into your internet browser https://swanseachhs.eu.qualtrics.com/jfe/form/SV_3K9uzs6aELpJJhc or by scanning the QR code below using your phone. Scanning the QR code should take you directly to the questionnaire.

All responses will be anonymous unless you choose to leave your contact details.

Before you continue, please tick the box to confirm you understand that the information you provide will be reviewed by the team of researchers conducting the evaluation and will be held securely by Swansea University ☐ **(*please tick)***

If you would like to speak to a member of the team about this research please contact:
Dr Sharon Parsons - S.N.Parsons@Swansea.ac.uk or Dr Liv Kosnes – L.Kosnes@Swansea.ac.uk

ABOUT THE APPOINTMENT:

1. **Name of your GP Surgery:** ____________________________________

2. **Month appointment took place:** _______________________________

 © SABU Consortium – not to be used without permission

3. **What type of appointment did you attend?** ***(please tick one box)***

   a. Face to face at your own GP surgery ☐

   b. Face to face at another venue ☐

   c. Video via a computer / tablet / smartphone ☐

   d. Telephone ☐

   e. Other ☐ *(please specify)* ________________________________

4. **Were you given a choice of how and when the appointment took place?** ***(Please tick)***

   Yes ☐ **If yes, was the surgery able to accommodate your preference?** Yes ☐ No ☐

   No ☐ **If no, would you have preferred a different type of appointment?** Yes ☐ No ☐

   Not sure ☐

5. **Thinking about the appointment, how satisfied were you with the following?** ***(Please tick)***

	**Not at all satisfied**	**Slightly satisfied**	**Moderately satisfied**	**Very satisfied**	**Completely Satisfied**
**Location of the appointment**					
**Time of the appointment**					
**Length of the appointment**					

6. **In your own words, what made you decide to attend your AWDPP appointment?**

THE INFORMATION YOU WERE GIVEN:

7. **Was the information provided at the appointment?** ***(Please tick)***

   Not enough ☐ Just right ☐ Too much ☐

 © SABU Consortium – not to be used without permission

8. **Thinking about the information leaflets you received either before or at your appointment, how useful did you find the information as a whole? *(Please tick)***

Extremely useful ☐ Very useful ☐ Moderately useful ☐ Slightly useful ☐ Not at all useful ☐

8a. Please tick below which leaflets you received, and how useful you found them:

**Leaflet**	**Yes, received**	**No, Did not receive**	**Do not recall**		**If received, how useful did you find it?**		
					**Very Useful**	**Somewhat Useful**	**Not at all Useful**
**AWDPP Information Leaflet**							
**Eatwell Guide**							
**Diabetes UK "Eating Well"**							
**Diabetes UK "Be Active"**							
**Let's Prevent Diabetes Flyer**							

8b. Please share any comments you have on the information received:

YOUR RISK OF DEVELOPING TYPE 2 DIABETES (T2DM)

9. **Before you were contacted about the All Wales Diabetes Prevention Programme (AWDPP), did you know you were at risk of developing Type 2 Diabetes (T2DM)? *(Please tick)***

Yes ☐ No ☐

 © SABU Consortium – not to be used without permission

10. How did you feel when you were first told you were at risk of developing T2DM?

11. Do you understand <u>YOUR</u> risk factors for developing T2DM? *(Please tick)*

Yes ☐ Somewhat ☐ No ☐

12. How important is it to you that you make lifestyle changes to reduce your risk of developing T2DM? *(Please tick)*

Extremely important ☐ Very important ☐ Somewhat important ☐ Slightly important ☐ Not at all important ☐

13. How confident do you feel about making lifestyle changes? *(Please tick)*

Extremely confident ☐ Very confident ☐ Somewhat confident ☐ Slightly confident ☐ Not at all confident ☐

<u>GOING FORWARD</u>

14. Were you offered any additional support at your AWDPP appointment, for example referral to another service? (e.g. Weight management service, Foodwise for Life, National Exercise Referral Scheme) *(Please tick)*

Yes ☐ ***(please give details in the box below)*** No ☐ ***(Go to Q15)***

14a. Do you plan to take up any of these offers of additional support?

Yes ☐ ***(Please give details in the box below)*** No ☐

 © SABU Consortium – not to be used without permission

14b. Is there anything making it difficult or stopping you from taking up the offers of support?

Yes ☐ ***(Please explain in the box below)*** No ☐

DEVELOPING THE PROGRAMME:

15. Overall, how useful did you find your AWDPP appointment? ***(please tick one box)***

Not at all useful ☐ Slightly useful ☐ Moderately useful ☐ Very useful ☐ Extremely Useful ☐

16. Are there any ways in which you feel the appointment could be improved?

Yes ☐ ***(please tell us how in the box below)*** No ☐

17. Some people who are invited to take part in the AWDPP do not attend their appointment. What do you think can be done to encourage people to attend the programme?

18. Is there anything else you would like to tell us about your experience of the AWDPP?

 © SABU Consortium – not to be used without permission

ABOUT YOU:

How would you describe your gender?

Please specify……………………………………………….. Prefer not to say ☐

What is your age?

18-24 ☐ 25-29 ☐ 30-34 ☐ 35-39 ☐ 40-44 ☐ 45-49 ☐

50-54 ☐ 55-59 ☐ 60-64 ☐ 65-69 ☐ 70-74 ☐ 75-79 ☐ 80+ ☐

Are you currently employed?

Yes, I work full time ☐ Yes, I work part time ☐ No, I am retired ☐ No, I am a carer ☐ No, I am in education / training ☐ No, I am not currently employed ☐

Do you consider yourself as having a disability?

Yes ☐ No ☐

What is your ethnic group?

White

British ☐ Irish ☐ Gypsy or Irish Traveller ☐ Any other white background ☐

Mixed/multiple ethnic groups

White and Black Caribbean ☐ White and Black African ☐ White and Asian ☐ Any other mixed / multiple ethnic background ☐

Asian/Asian British

Indian ☐ Pakistani ☐ Bangladeshi ☐ Chinese ☐ Any other Asian background ☐

Black/African/ Caribbean/ Black British

African ☐ Caribbean ☐ Any other Black / African / Caribbean background ☐

Other ethnic group

Arab ☐ Any other ethnic group ☐

<u>THANK YOU FOR TAKING THE TIME TO COMPLETE THIS QUESTIONNAIRE</u>.

<u>Please return it in the FREEPOST envelope provided</u>

 © SABU Consortium – not to be used without permission

WOULD YOU LIKE TO TAKE PART IN FURTHER RESEARCH TO SHAPE THE DEVELOPMENT OF THE ALL WALES DIABETES PREVENTION PROGRAMME?

We will be holding a small number of focus groups and interviews with people who have taken part in the AWDPP to get a better understanding of their experiences and views on how the programme could develop. The focus groups and interviews will either take place face-to-face in a local location, virtually or via telephone (interviews only). Travel expenses will be provided where appropriate.

We will be holding one focus group in each Health Board area and up to 10 interviews across Wales. We are keen to speak to people from a wide variety of backgrounds and may use the information you have provided in the 'About You' section to ensure we have wide representation in the focus groups and interviews. We may not be able to speak to all those who volunteer in detail but will acknowledge your interest and be in contact with further information if you have been selected to take part in the research.

If you would be interested in helping in this way, please provide your contact details below:

Name:

Contact details:
(either an address, email or phone number)

 © SABU Consortium – not to be used without permission

 © SABU Consortium – not to be used without permission
