# Supplementary material for: The challenges and lessons from a formative process and value-based evaluation of the wave 1 roll-out of the all Wales Diabetes Prevention Programme
Source: BMC Public Health. 2024 Sep 13;24:2499. doi: 10.1186/s12889-024-19946-0 (PMC11401378; doi:10.1186/s12889-024-19946-0)
Supplement: Supplementary file 6 — Supplementary Material 6. General Practice Questionnairepdf fileGeneral Practice QuestionnaireGeneral Practice questionnaire. [file 12889_2024_19946_MOESM6_ESM.pdf]

All Wales Diabetes Prevention Programme Process Evaluation

General Practice Questionnaire

You are being invited to take part in the '***Formative Process and Value Based Evaluation for the All Wales Diabetes Prevention Programme (AWDPP)***'. The evaluation is being conducted by a collaboration of researchers from Swansea, Aberystwyth and Bangor Universities on behalf of Public Health Wales.

Contributing to the Healthy Weight: Healthy Wales Delivery Plan 2021-22, Welsh Government is funding a national two-year roll-out of a Type 2 Diabetes (T2DM) intervention, under the All Wales Diabetes Prevention Programme (AWDPP). The AWDPP has been developed by a group of healthcare professionals and scientists in Wales to provide a lifestyle intervention to people at risk of developing T2DM to see if it can help to prevent or delay the onset of the condition.

The AWDPP is led by the Primary Care Division in Public Health Wales, and in partnership with the seven Health Boards across Wales. The national roll out of the intervention will be delivered through primary care clusters within each of the seven Health Boards.

The ***Process and Value Based Evaluation*** will look at how the AWDPP is being delivered and will examine both patient and healthcare professionals' perspectives.

As a General Practice participating in the first wave of the Welsh Government funded rollout, we would be grateful if your practice could share your experience of the AWDPP and any impact it has had so far.

Before you continue, please tick the box to confirm you understand that the information you provide will be reviewed by the team of researchers conducting the evaluation and will held securely by Swansea University ☐ **(*please tick)***

Name of Practice: ________________________________

Your Role within the Practice: GP ☐

GP Lead for Diabetes ☐

Practice Manager ☐

Practice Nurse ☐

Other *(please specify)* __________________________

 © SABU Consortium – not to be used without permission

1. How informed do you feel about the All Wales Diabetes Prevention Programme (AWDPP) and the intervention that is being used?

Extremely informed ☐	Very informed ☐	Somewhat informed ☐	Slightly informed ☐	Not at all informed ☐

2. What is your involvement in the AWDPP?

3. Would you like to be more involved in the AWDPP?

Yes ☐ No ☐ (go to Q4)

If yes, how would you like to be more involved?

4. Do you think the programme is benefitting patients in your practice who are at risk of developing T2DM?

Yes ☐ If yes, how do you feel patients are benefitting? *(explain below)*

No ☐ If no, why do you think patients are not benefitting? *(explain below)*

Not sure ☐

5. Do you think the inclusion criteria are capturing all the people who would benefit from an intervention aimed at people at risk of T2DM?

Yes ☐

No ☐ If no, who do you think would benefit from being invited ? *(explain below)*

Not sure ☐

 © SABU Consortium – not to be used without permission

6. Have there been any unexpected outcomes as a result of the practice being involved in the AWDPP?

Yes ☐ *(Please give details below)* No ☐

7. What factors have enabled the practice to implement the AWDPP?

8. Have there been any barriers to the practice implementing the AWDPP?

Yes ☐ *(Please give details below)* No ☐

9. On a scale of 1 to 10, how important do you think T2DM prevention work is? *(please circle)*

1	2	3	4	5	6	7	8	9	10
Not at all important									Extremely important

10. Which teams do you think are most appropriate to carry out T2DM prevention work?

(Select all that apply)

General Practice ☐

Public Health Wales ☐

Community Support Teams (e.g. Health & Wellbeing Facilitators, Inverse Care Law Team) ☐

Dietetics / Weight Management Teams ☐

Specialist Diabetes Teams based in Primary Care ☐

Specialist Diabetes Teams based in Secondary Care ☐

Third Sector Organisations (e.g. Diabetes UK) ☐

Other *(Please specify)* ______________________________

 © SABU Consortium – not to be used without permission

11. Other than the AWDPP, are you currently involved in T2DM prevention / prediabetes work at your practice?

Yes ☐ *(Please give details below)* No ☐

12. Going forward, are there any barriers to continuing to deliver the AWDPP in your practice?

Yes ☐ *(Please give details below)* No ☐

13. How confident are you that your practice will be able to allocate time and resources to T2DM prevention / prediabetes work going forward?

Extremely confident ☐ Very confident ☐ Somewhat confident ☐ Slightly confident ☐ Not at all confident ☐

14. Has being involved in the AWDPP changed the way you plan to undertake diabetes prevention and manage people at risk of T2DM in your practice?

Yes ☐ No ☐ Partially ☐

Please explain below:

15. Please use this space to make any further comments you may have about the implementation of the All Wales Diabetes Prevention Programme?

Thank you for your time

 © SABU Consortium – not to be used without permission
