# Supplementary material for: The challenges and lessons from a formative process and value-based evaluation of the wave 1 roll-out of the all Wales Diabetes Prevention Programme
Source: BMC Public Health. 2024 Sep 13;24:2499. doi: 10.1186/s12889-024-19946-0 (PMC11401378; doi:10.1186/s12889-024-19946-0)
Supplement: Supplementary file 7 — Supplementary Material 7. Primary Care Cluster Demographicspdf filePrimary Care Cluster DemographicsTable of primary care cluster area demographic details. [file 12889_2024_19946_MOESM7_ESM.pdf]

Primary Care Cluster Demographics

Practice population data was provided by NHS Wales Shared Services Partnership and is a snapshot taken at a single point in time. The time stamp for the data used was January 2022. The metadata description of the data set notes the resident lower layer super output areas (LSOAs) of service users registered to each general practice were matched to the Welsh Index of Multiple Deprivation (WIMD) 2019 to count the number of patients who live in the most deprived 20% of LSOAs as determined by their WIMD ranking. The metadata also notes that general practice data was aggregated to primary care clusters and rankings were created based on two measures to estimate deprivation at primary care cluster level. These were:

1) The number of patients registered to the general practice within the cluster who live in the most deprived 20% of areas in Wales.
2) The percentage of each cluster population that live in the most deprived 20% of areas in Wales.

The burden of disease for diabetes per primary care cluster refers to the prevalence and impact of diabetes on the population in different geographic areas served by general practitioner (GP) practices in Wales. It includes the number of people diagnosed with diabetes, the severity of the disease, and the associated health outcomes, such as hospitalizations, complications, and premature deaths.

Ethnicity data were aggregated to primary care clusters from ONS census data (2021).

Estimates of the percentage of the registered practice population (aged 16+) that are overweight or obese, as recorded by the Welsh Health Survey. A person is described as overweight if their BMI is 25+ and obese if their BMI is 30+ (Public Health Observatory, 2014).

Health Board	Aneurin Bevan University Health Board		Betsi Cadwaladr University Health Board		Cardiff and Vale University Health Board		Cwm Taf Morgannwg University Health Board			Hywel Dda University Health Board		Powys Teaching Health Board		Swansea Bay University Health Board	
**Primary Care Cluster**	**Blaenau Gwent West**	**Caerphilly North**	**Anglesey**	**Meirionnydd**	**Central Vale**	**City & Cardiff South**	**Bridgend West**	**Merthyr North and South**		**North Ceredigion**	**South Ceredigion**	**Powys -Mid**	**Powys -North**	**City Health**	**Upper Valleys**
**No of practices**	5	8	10	6	7	6	3	7		7	5	5	7	8	4
**No of fully qualified GPs FTE**	20.6	28.2	37.5	20.7	31.3	19.0	11.2	17.0	13.6	22.0	24.1	16.2	33.2	27.1	16.4
**No of nurses FTE**	12.3	21.0	28.3	10.7	18.8	6.3	6.5	8.4	6.7	25.1	13.1	9.0	34.4	14.1	14.6
**Cluster population (N)**	38777	63416	65884	19130	65492	41942	34610	35420	25749	45352	47210	28429	63921	51905	31534
**% Population aged 65+**	19.7%	19.9%	23.6%	27.1%	17.3%	10.7%	24.2%	16.2%	18.2%	21.7%	25.9%	28.5%	24.2%	16.9%	21%
**Ethnicity profile of cluster population (percentage white)**	97.9%	98.0%	97.7%	98.3%	94.8%	57.6%	97.8%	96.9%	98.0%	95.4%	97.9%	98.1%	98.2%	84.2%	98.2%
**Cluster obesity prevalence (BMI >25) 2014**	60.7%	60.1%	59.5%	58.4%	58.1%	58.5%	58.7%	59.9%	59.4%	52.4%	59.4%	58.6%	58.2%	59.1%	59.4%
**Cluster diabetes prevalence 2019/2020**	9.4%	9.1%	8.7%	8.9%	7.4%	7.1%	8.4%	8.8%	7.9%	6.6%	8.4%	7.9%	7.7%	7.4%	8.5%

Primary Care Cluster	Blaenau Gwent West	Caerphilly North	Anglesey	Meirionnydd	Central Vale	City & Cardiff South	Bridgend West	Merthyr North and South		North Ceredigion	South Ceredigion	Powys -Mid	Powys -North	City Health	Upper Valleys
**Cluster recorded burden of diabetes disease (2016)***	8.3%	8.6%	7.3%	7.6%	6.7%	6.2%	7.5%	7.0%	7.5%	5.8%	7.1%	7.5%	6.8%	7.1%	8.0%
**Number and percentage of service users living in most deprived 20% of WIMD**	15256 (39.3%)	23309 (36.8%)	9746 (14.8%)	0	16163 (24.7%)	21366 (50.9%)	3882 (11.2%)	13973 (39.4%)	4937 (19.2%)	<5 (0.002%)	2357 (5%)	1320 (4.6%)	5723 (9%)	24306 (46.8%)	1799 (5.7%)
**Cluster deprivation quintile**	1	2	3	5	2	1	3	1	3	3	5	4	5	1	4
