# Supplementary material for: The challenges and lessons from a formative process and value-based evaluation of the wave 1 roll-out of the all Wales Diabetes Prevention Programme
Source: BMC Public Health. 2024 Sep 13;24:2499. doi: 10.1186/s12889-024-19946-0 (PMC11401378; doi:10.1186/s12889-024-19946-0)
Supplement: Supplementary file 8 — Supplementary Material 8. Service User Survey Demographicspdf fileService User Survey DemographicsTable of service users demographic details. [file 12889_2024_19946_MOESM8_ESM.pdf]

**Demographic details of those who completed the Service User Survey**

| | N | % |
|---|---|---|
| ***Gender*** | | |
| Male | 51 | 44% |
| Female | 50 | 43% |
| Prefer not to say | 2 | 2% |
| Missing | 13 | 11% |
| ***Age (years)*** | | |
| 40-44 | 2 | 2% |
| 45-49 | 1 | 1% |
| 50-54 | 3 | 3% |
| 55-59 | 13 | 11% |
| 60-64 | 8 | 7% |
| 65-69 | 38 | 33% |
| 70-74 | 16 | 14% |
| 75-79 | 26 | 22% |
| Missing | 9 | 8% |
| ***Employment*** | | |
| Not currently employed | 10 | 9% |
| Full-time carer | 2 | 2% |
| Retired | 82 | 71% |
| Part- time employed | 6 | 5% |
| Full-time employed | 13 | 11% |
| Missing | 3 | 3% |
| ***Ethnicity*** | | |
| White British | 106 | 91% |
| White Irish | 1 | 1% |
| Chinese | 1 | 1% |
| Any other Asian origin | 1 | 1% |
| Black African | 2 | 2% |
| Arabian | 1 | 1% |
| Missing | 4 | 3% |
| ***Disability*** | | |
| Yes | 21 | 18% |
| No | 90 | 78% |
| Missing | 5 | 4% |
